# Stereomicroscopic evaluation of sealing ability of four different root canal sealers: an in-vitro study

**DOI:** 10.1186/s12903-024-03913-7

**Published:** 2024-02-20

**Authors:** Sonam Sah, Panna Mangat, Ajay Kumar, Neha Sah, Ganiga Channaiah Shivakumar, Marco Di Blasio, Gabriele Cervino, Giuseppe Minervini

**Affiliations:** 1 Department of Conservative Dentistry and Endodontic Teerthanker Mahaveer Dental College and Research Centre Moradabad, Moradabad, UP India; 2 Department of Conservative Dentistry and Endodontics Kalka Dental College and Hospital, Meerut, UP India; 3 Unit of Oral Medicine and Radiology, Faculty of Dental Sciences, I.M.S, B.H.U. Varanasi, Varanasi, UP India; 4 Unit of oral and maxillofacial surgery, faculty of dental sciences, I.M.S, B.H.U. Varanasi, Varanasi, UP India; 5 Department of Oral Medicine & Radiology, Peoples College of Dental Sciences, Peoples University, Bhanpur, Bhopal, Madhya Pradesh India; 6 Department of Medicine and Surgery, University Center of Dentistry, University of Parma, Parma, 43126 Italy; 7 School of Dentistry, Department of Biomedical and Dental Sciences and Morphofunctional Imaging, University of Messina, via Consolare Valeria, 1, Messina, 98125 Italy; 8 Saveetha Dental College and Hospitals, Saveetha Institute of Medical and Technical Sciences (SIMATS), Saveetha University, Chennai, Tamil Nadu India; 9 Multidisciplinary Department of Medical-Surgical and Dental Specialties, University of Campania Luigi Vanvitelli, Naples, 80121 Italy

**Keywords:** Root canal sealers, Stereomicroscopic evauation, Endodontic

## Abstract

**Aim:**

To compare and evaluate the sealing ability of four different commercially available sealers to provide seal against the dye penetration test using a stereomicroscope-an in-vitro study.

**Material/Method:**

80 extracted single rooted mandibular premolar with single canal were used in this study. The samples were divided in 4 groups (20 in each) based on sealer. Group I (Diaproseal), Group II (apexit Plus), Group III (MTA Fillapex) and Group IV (Bio-C). The samples were analyzed using a stereomicroscope and data analysis was done with one-way Anova And post hoc Tukey’s test.

**Result:**

The mean dye penetration score was 1.2400 ± 0.778 mm for Group I. 2.6000 ± 0.897 mm for Group II, 4.2000 ± 0.923 mm for Group III and 4.225 ± 2.055 mm for Group IV. One-way Anova analysis shows that intergroup comparison was statistically significant between the four groups. The post hoc Tukey’s test reveals that the difference was statistically non-significant between group III and group IV.

**Conclusion:**

It was concluded that between the four groups the Group I (Diaproseal) showed the least dye penetration followed by Group II (Apexit Pus), Group III (MTA Fillapex) and then Group IV (Bio-C), where there was no significant difference between the Group III (MTA Fillapex) and Group IV (Bio-C).

## Introduction

According to Schilder H. 1967 [1] in his study, filling root canals in three dimensions in the final analysis he concluded that it is the sealing off of the root canal system from the periodontal ligament and the bone which ensure the health of the attachment apparatus against the breakdown of the endodontic origin. There are different terms used to describe the seal of the root canal system. A proper term that should be use is a “Fluid-Tight seal” or “Fluid Impervious seal” because the seal of the root canal are commonly evaluated against fluid leakage – a parameter used to approve or disapprove obturation materials and techniques [2–4]. The role of root canal sealer along with the gutta percha is crucial to fill the interface between the dentin wall and obturating material to bring off this fluid tight seal as the sealer contacts the root canal wall, flow into the complex anatomy of root canal system like accessory and lateral canal, voids, spaces, isthmus, deltas and also penetrates into the dentinal tubules. There have been many types of sealers that are used with the gutta percha to obturate the root canal and recent advances being MTA and Bioceramic.

Calcium hydroxide-based root canal sealer (Ivoclar Vivadent Apexit® plus), has been introduced in an attempt to provide a flawless seal at the apical foramen without damaging periodontal tissues [5]. The high pH provided by this sealer (to above 12.5) may be responsible for its antimicrobial effect [6–11]. Recently, a new root canal sealer Dia-ProSeal (DiaDent, Cheongju, Korea 2014) has been introduced to substitute conventional sealers with the guarantee of improved clinical performance [12–17]. However, the Resin based sealers have disadvantage of polymerisation shrinkage. So recently MTA based root canal sealers have come as a favourable and bioactive alternative. MTA Fillapex (Angelus Londrina/Parana/Brazil 2010) a new sealer marketed recently, claim to have alkaline pH and subsequent antibacterial activity. MTA Fillapex is first paste: paste MTA- based salicylate resin root canal sealer and has a high flow rate (27 mm) and a low film thickness [18–20]. Bio C sealer (Angelus BIO-C® Sealer 2010) is another new, premixed, ready-to-use bioactive, Bio-ceramic based root canal sealer, available in a single syringe. Its bioactivity is claimed to be because of the release of calcium ions that stimulating the formation of mineralized tissue through bioconductivity [21]. 

In dentistry, a variety of materials are used to restore teeth and treat dental issues. Amalgam, a traditional material for fillings, is known for its strength and longevity. Composite resins are aesthetic materials that match the natural tooth color, favored for both anterior and posterior restorations [21]. Ceramics, including porcelain, offer superior aesthetics for crowns and veneers, while advanced ceramics like zirconia provide exceptional durability. Glass ionomer cements release fluoride and are ideal for non-load bearing areas due to their weaker structure. Gold alloys are less common now but are valued for their durability and biocompatibility in crowns and bridges. Base metal alloys, such as nickel-chromium, are cost-effective alternatives for prosthetics. Polymers, like PMMA, are primarily used for dentures. Moreover, endodontic materials such as gutta-percha are used to fill and seal the root canal after treatment. Each material is chosen based on the specific needs of the tooth restoration or treatment [22–25].

Micro leakages have shown their deleterious effect on the success of endodontic treatment. One of the major causes of the failure of root canal treatment is incomplete obturation of the root canal space that allows the penetration of micro-organism and their toxins products through the unfilled spaces or from space created by degradation of the sealer that may remain active in the dentinal tubules even after vigorous irrigation of the root canal system during chemico-mechanical preparation. Thus, perfect apical sealing is desirable to prevent the remaining bacteria and their endotoxins from reaching the root apex [22–25]. 

The most common technique for evaluating the root canal sealer sealing ability is the dye penetration method [26, 27]. So this study was aimed at evaluating the sealing ability of four different commercially available sealers using a stereomicroscope analysis of dye penetration.

## Material & method

A total of 80 extracted single rooted permanent mandibular premolars with a single root canal were selected for this study. The study was approved by the institutional ethical review board of Dental College and Research Centre (IERB) with reference number KDC/IES/2019/0176, dated 22/11/2019 and followed all the recommendation of Helsinki declaration. Exclusion criteria included tooth with carious lesion, fractured root, evidence of craze line, evidence of any resorption and incomplete apex formation.

### Preparation of teeth

**Fig. 1:**
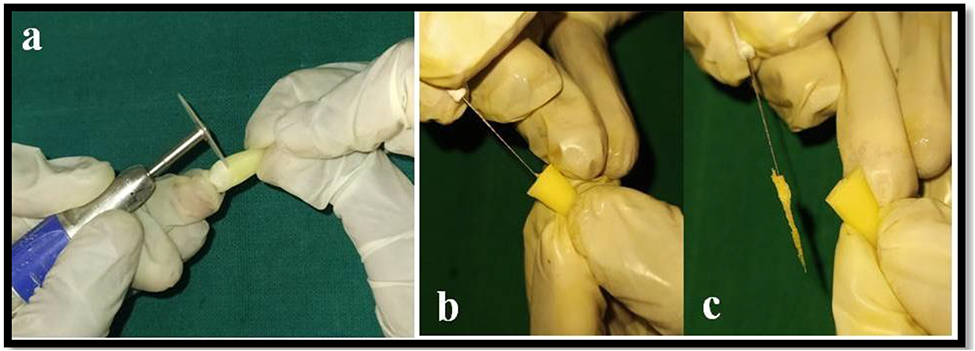
Showing **a**) Decoration **b**) & **c**) Pulp extirpation

Samples were cleaned of any visible debris, tissue remnants & calculus and placed in 5.25% sodium hypochlorite for 2 h & then stored in a normal saline until further use. The crowns of all the teeth were sectioned at the level of Cementoenamel Junction with a diamond disc (Fig. 1a). Removal of pulp tissue was done with a barbed broach (Fig. 1b and c) and patency of the canals was checked with #10 k-type file.

**Fig. 2:**
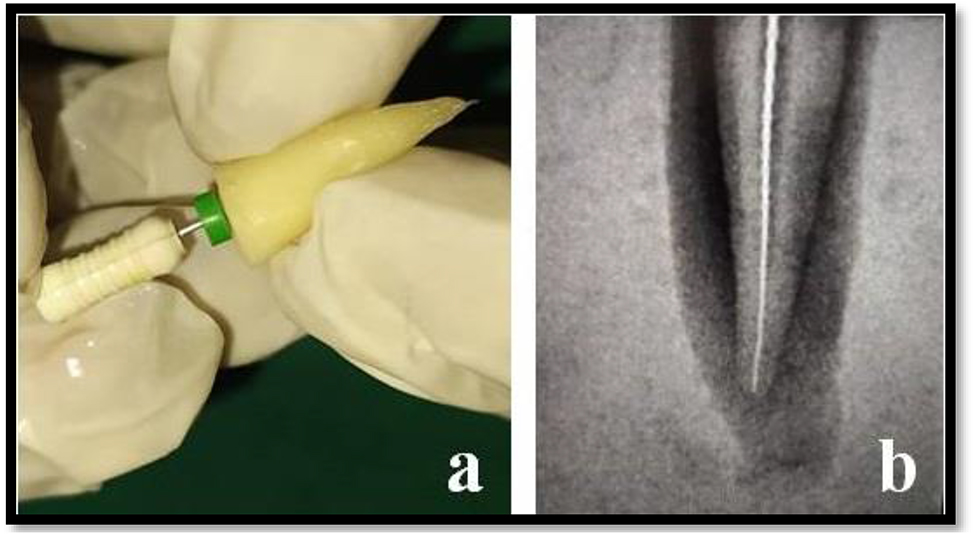
**a**) & **b**) Working length determination

To determine the working length #15 K-type file was inserted into the root canals until the instrument tip was visible from the apex and this length was then recorded. 1 mm was subtracted from this recorded length and the working length was determined (Fig. 2a and b).

**Fig. 3:**
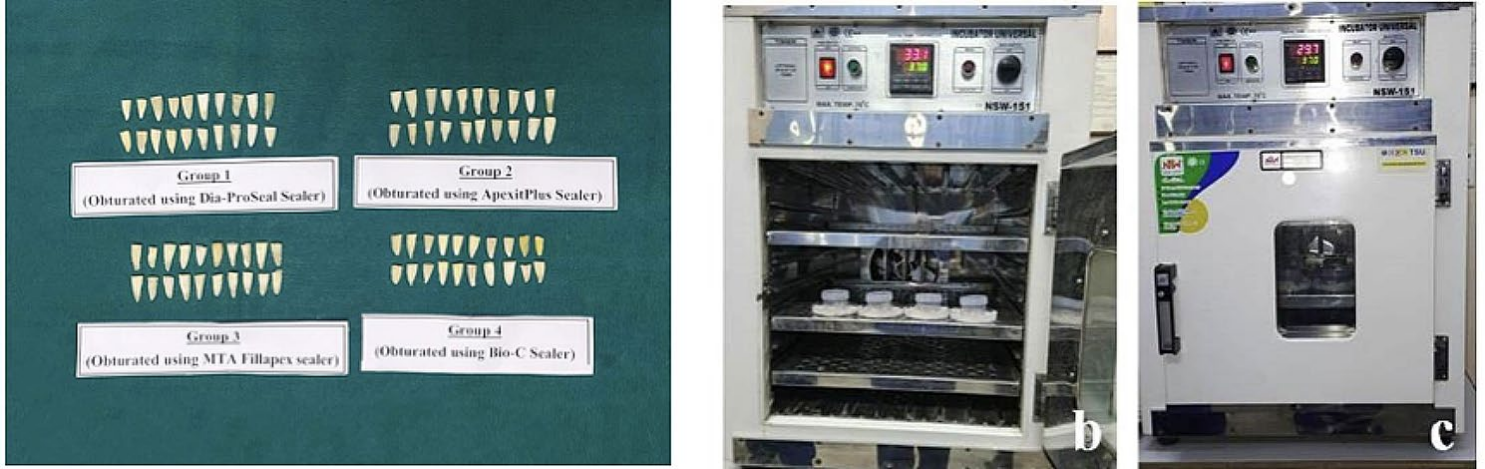
**a**) Grouping of samples based on sealer used. **b**) & **c**) Samples placed in incubator cone fit

Chemico-mechanical preparation was done with crown down technique using ProTaper Gold rotary instruments upto F3 file size. #10 k-type file was passed 1 mm through the apical foramen to remove any dentinal plug and ensure the patency of the apical foramen for dye penetration. In between each instrumentation canals were irrigated using 5.25% sodium hypochlorite and 17% EDTA followed by final irrigation with normal saline and then, the canals were dried using absorbent paper points. The F3 ProTaper gutta-percha was selected for each canal. Teeth were chosen at random for this level and grouped into four groups of 20 teeth each. The four experimental group received gutta-percha along with different types of sealer (Fig. 3a, b and c).

**Fig. 4:**
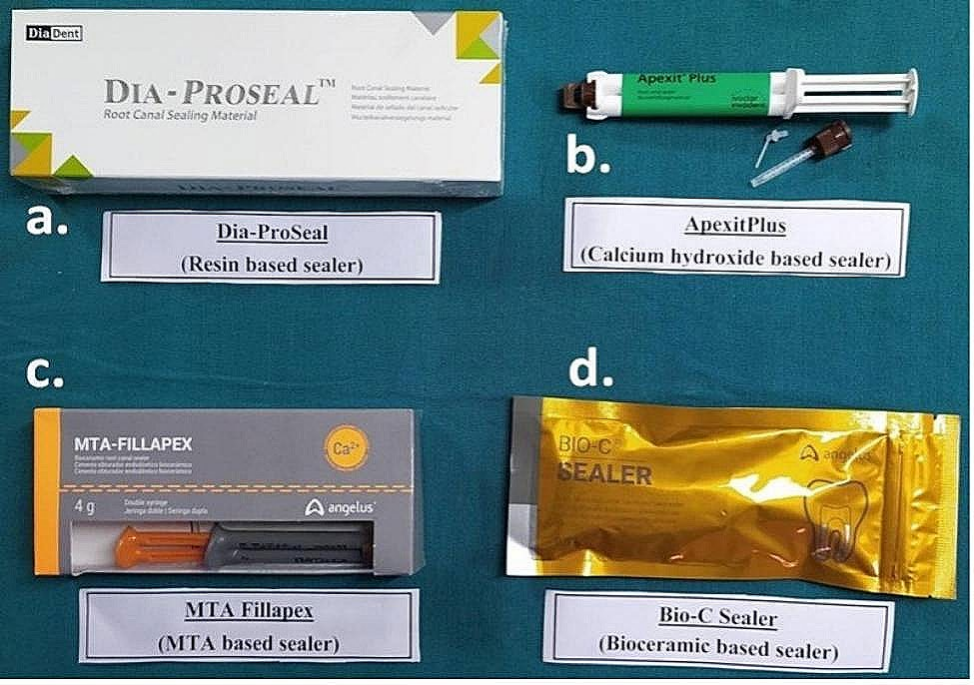
**a**) Dia-Proseal Sealer, **b**) Apexit Plus Sealer, **c**) MTA Fillapex sealer & **d**) Bio-C Sealer

**GROUP 1** – Gutta-percha with Resin based sealer, Dia-Proseal (Fig. 4a).

**GROUP 2** – Gutta-percha with Calcium hydroxide-based sealer, Apexit Plus (Fig. 4b).

**GROUP 3** – Gutta-percha with MTA based sealer, MTA Fillapex (Fig. 4c).

**GROUP 4** – Gutta-percha with Bio-ceramic sealer, Bio-C (Fig. 4d).

**Fig. 5:**
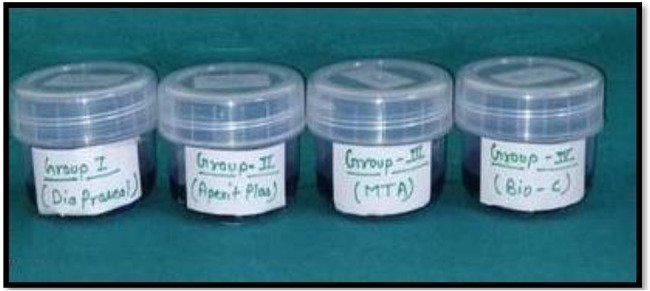
Samples immersed in dye

Mixing of sealer was done according to manufactures direction and introduced into each canal using a lentulo-spiral paste carrier. Then the master cone F3 gutta-percha points were coated with the sealers and placed in canals till full working length. Access cavities were sealed using a temporary restorative material and all the samples were placed in an incubator (NSW) for two weeks with 100% humidity at 37^0^ C (Fig. 3b & c). After humidification was done, all the teeth were coated with 2 layer of nail varnish except for the apical 3 mm and immersed in freshly prepared 1% methylene blue dye for 72 h (Fig. 5) and then bathed in running tap water.

**Fig. 6:**
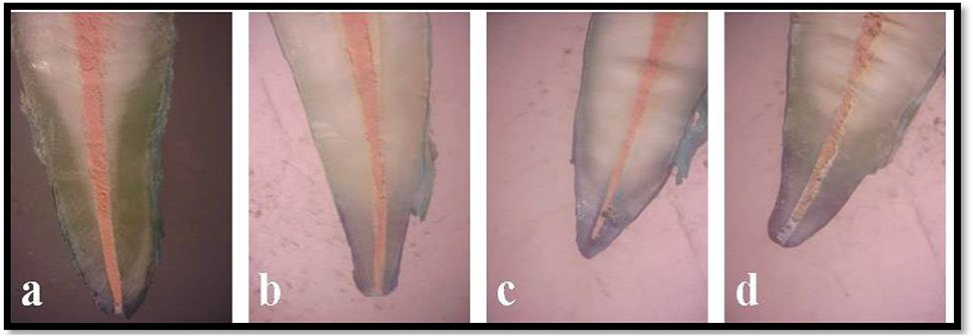
Stereomicroscopic images of **a**) Group I, **b**) Group II, **c**) Group III and **d**) Group IV

The samples were sectioned longitudinally roughly parallel to the long axis of the tooth and through the apex. The samples were then studied under a stereomicroscope (ALCO®) with camera (Olympus) at x10 magnification (Fig. 6) to observe the measurement of dye penetration from apex to the end point of dye penetration. To evaluate the apical leakage in this in vitro study, Escobar’s [28] criteria were used to evaluate the infiltration proportions:

**Graph 1:**
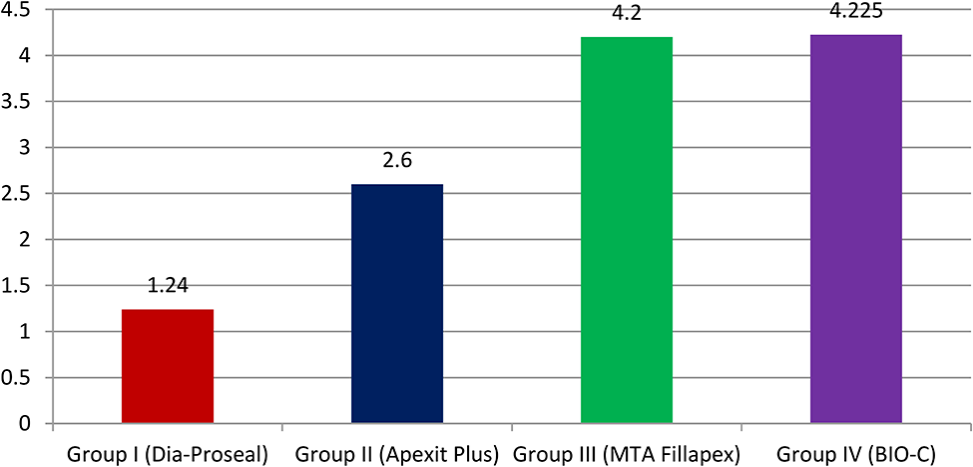
Mean measurements of dye penetration in Group I, Group II, Group III and Group IV

0. Infiltration loss (dye penetration 0–<1.5 mm).
1. Simple infiltration (dye penetration 1.5–3 mm).
2. Medium infiltration (dye penetration > 3 mm).

## Results

Based on the dye penetration scores 65% of the subjects were having score 0 in the Group I, 75% were having score 1 for the Group II, 75% were having score 2 in the Group III and 55% were having score 2 in the Group IV and 35% were having score 1 in the Group IV (Table 1). The mean dye penetration scores were 1.2400 ± 0.778 for the Group I, 2.6000 ± 0.897 for the Group II, 4.2000 ± 0.923 for the Group III, 4.225 ± 2.055 for the Group IV (Graph 1). The post hoc analysis revealed significant difference between Group I and Group II, Group I and Group III, Group I and Group IV, Group II and Group III, Group II and Group IV and the difference was statistically non-significant between Group III and Group IV (Table 2). The intergroup comparison was statistically significant between the four groups when analyzed using the One Way ANOVA at p value less than 0.001 (Tables 3 and 4) (Fig. 6a,b,c,d).

**Table 1:** Dye penetration score based on Escobar’s scoring criteria

Groups	Score 0	Score 1	Score 2	Chi-square value	p-value
Group I (Dia-Proseal)	13 (65%)	5 (25%)	2 (10%)	52.055	0.001
Group II (Apexit Plus)	0 (0%)	15 (75%)	5 (25%)		
Group III (MTA Fillapex)	0 (0%)	5 (25%)	15 (75%)		
Group IV (KIO-C)	2 (10%)	7 (35%)	11 (55%)		

**Table 2:** Intergroup Post-Hoc analysis

Comparison	Mean difference	P value	Significance
Group I vs. Group II	1.360	0.001	Significant
Group I vs. Group III	2.960	0.001	Significant
Group I vs. Group IV	2.985	0.001	Significant
Group II vs. Group III	1.600	0.001	Significant
Group II vs. Group IV	1.625	0.001	Significant
Group III vs. Group IV	0.025	0.951	Non-Sig

**Table 3:** Intergroup comparison using one way ANOVA

Group	Mean	Std. Deviation	Std. Error	Minimum	Maximum	P value
Group I (Dia-Proseal)	1.2400	0.77826	0.17402	0.50	3.50	
						0.001 Significant
Group II (Apexit Plus)	2.6000	0.39736	0.20066	1.50	4.00	
Group III (MTA Fillapex)	4.2000	0.92338	0.20647	3.00	6.00	
Group IV (KIO-C)	4.2250	2.055	0.45951	1.50	8.00	

**Table 4:** Abbreviations used in the study

Abbreviation	Full Name
Dia-ProSeal	Dia-ProSeal sealer
AH Plus	AH Plus sealer
MTA Fillapex	MTA Fillapex sealer
Bio-C	Bio-C sealer
ZOE	Zinc oxide eugenol sealer
RSA	Roekoseal Automix sealer
MTA	Mineral trioxide aggregate
ISO	International Organization for Standardization
OHˉ	Hydroxide ion
Ca2+	Calcium ion
Bioroot RCS	Bioroot RCS sealer
Nanoseal S	Nanoseal S sealer
Eposeal	Eposeal sealer
IERB	Institutional ethical review board
EDTA	Ethylenediaminetetraacetic acid
NSW	Normal saline

## Discussion

The Microleakage in the form of bacteria and its byproducts, fluids and chemical substances inside the root canal system is one of the causes of endodontic therapy failure. The aim of obturation is to eliminate pathways of leakage from the apical and coronal directions and also to entomb the remaining bacteria usually present in the dentinal tubules [29]. Though gutta percha is considered the most common root canal filling material worldwide, they do not provide complete sealing of root canal system as they do not adhere to dentine wall owing to its poor sealing properties [30–34]. A root canal sealer applied to the dentinal walls of a canal in order to fill the irregularities between the obturating material and the canal walls, thus providing a fluid tight seal [35]. The dye penetration technique along with stereomicroscopic evaluation is most commonly employed method for assessment of apical microleakage because of the simplicity of laboratory procedure and final reading of the results [36, 37]. Numerous brands and types of root-canal sealers are developed in current endodontic practices.

In this study Dia-ProSeal sealer (new, resin based), showed significantly lowest dye penetration than other sealers. It have various properties such as good sealing ability of complex root canal anatomies, fast setting time, dual syringe allowing easy mix, stability of volume and long term storage ability [38]. Song YS et al. (2016) [12] in their study compared Dia-ProSeal sealer with AH Plus and AD seal and concluded that Dia-ProSeal sealer have many valuable properties such as biocompatibility because of high pH range about 6.7–7.2 and less solubility of about 0.5 × 10^[−4]^ and better sealing ability because of adequate flow with acceptable physiochemical properties and dimensional changes about 0.5%. This can explain the lowest dye leakage of DiaProseal in our study as it has adequate flow to penetrate and seal the dentinal tubules. The lesser leakage with resin-based sealer can also be explained as the epoxy resin-based sealers are thought to be able to bond chemically to root dentin by reacting with any exposed amino groups in collagen and forms a covalent bond, thus having the higher bonding to dentin [39–41]. As they have good penetration ability into canal irregularities because of their long setting time and creep capacity, it also increases the mechanical interlocking between the root dentine and sealer [42]. 

Apexit® Plus a calcium hydroxide-based root canal sealer which may have good sealing ability by stimulating the deposition of hard tissues at the root apex and biocompatibility with tissues [43]. In this study Apexit Plus has showed significantly less dye penetration than MTA Fillapex and Bio-C sealers. The good sealing ability of calcium hydroxide-based sealer might be related to the alkaline pH of calcium hydroxide that activates alkaline phosphatase that plays an important role in hard tissue formation [39]. While the increased leakage in comparison to DiaProseal would be possible due to dissolution over the time and volumetric expansion during the setting period and a post setting period up to 21 days (Caicedo & Von Fraunhofer, 1988) [44]. As calcium hydroxide-based sealers are soluble and that quality could probably cause a deficiency in their sealing ability over an extended period of time [45, 46]. Patni PH et al. (2016) [47] compared effectiveness of apical seal obtained by ZOE, AH Plus, Apexit Plus and RSA sealers and found that Apexit Plus had significantly lesser dye penetration than ZOE sealer but higher than AH Plus and RSA.

MTA Fillapex showed high dye penetration than Apexit Plus and DiaProseal but lower when compared to Bio-C sealer, which showed the highest dye penetration although it was statically non-significant. Khade RK et al. (2021) [48] compared MTA Fillapex and AH plus sealer and the result demonstrated significant less dye leakage for AH Plus compared to MTA Fillapex. While in another study by Altan H et al. (2018) [49] at 24 h evaluation MTA Fillapex presented significantly less microleakage than Sealapex and AH Plus sealer but Sealapex and AH Plus presented significantly less microleakage than MTA Fillapex at long term interval of 180 days. The sealing ability of MTA Fillapex can be explained because of its composition as it contain salicylate resin and natural resin in its composition which increases the flow of the material to penetrate the dentinal tubules [40, 42, 50] and encourage apatite like crystalline deposits along the apical and middle third of root canal forming a interfacial layer with tag-like feature but the low adhesion capacity of these tag-like structures, result in the low bond strength of MTA Fillapex [51, 52]. The low bond strength will result in lesser sealing ability and thereby explaining the result of this study. Bio-C is a novel bioceramic, non-resin which stimulates tissue regeneration^19^ and the only ready-to-use cement with Tricalcium aluminates, providing the same biological interaction as MTA [53, 54] although with improved manipulation and insertion, known to induce osteo-promotive and bone-remembering and thought to contribute to the mineralization process of the periapical tissue [55–58]. They are biocompatible have appropriates setting time, flow and radiopacity besides alkalinization capacity reaching pH of 10 in 21 days [56, 59]. Solubility indicates the loss of material mass when immersed in water. In a study by Zordan-Bronzel CL et al. (2019) [60] who evaluated the physiochemical properties of new calcium silicate-based sealer, Bio-C, Bio-C sealer had shown higher solubility (17.9% ± 2.5%) than the rates required by ISO 6876 standard (< 3%). Calcium silicate–based sealers have shown high solubility after immersion in water compared with the standard resin-based sealers that can be explained by the hydrophilic nanosized particles that increase their surface area and allow more liquid molecules to come in contact with the sealer [56, 60]. Instead of showing high flow rate and shortest setting time, the high solubility of Bio-C sealers after immersion in water, may explain the highest dye penetration result in our study. Although the high solubility of calcium silicate–based sealers can be considered a disadvantage, their bioactive potential is a consequence of the solubility of these materials even after setting. Moreover, the solubility of calcium silicate–based sealers can be explained by the release of OH ˉ and Ca 2 + ions. An alkaline environment may play a positive role in apical healing, thus contributing to the formation of mineralized tissues [60]. 

Rashid et al. [61] evaluated the sealing ability of three commercially available endodontic sealers: Bioroot RCS (tricalcium silicate-based), Nanoseal S (polydimethylsiloxane-based), and Eposeal (epoxy resin-based). They found that Eposeal exhibited the least dye penetration, followed by Bioroot RC, and Nanoseal S showed the highest dye penetration. Thakur et al. [62] compared the apical sealing ability of four endodontic sealers: conventional zinc oxide eugenol sealer, Apexit, AH-Plus, and Roekoseal Automix (RSA). RSA, a polydimethylsiloxane-based sealer, demonstrated significantly better apical sealing compared to the other sealers. Pallavi et al. [63] investigated the microleakage of two endodontic sealers, AH Plus and MTA Fillapex, placed using two different techniques: master gutta-percha cone and size 30 lentulospiral. MTA Fillapex placed using lentulospiral achieved the highest apical seal among the experimental groups.

However, till date, no sealer has been shown to be totally satisfactory for clinical use. The materials that have been used for obturation of root canal system, they have their own advantages and disadvantages and there is no single material or technique available so far, that fulfill all the requirement of root canal obturation.

## Conclusion

Within the limitation of this study following conclusion was drawn;

- The Diaproseal sealer showed the minimal dye penetration and Bio-C sealer showed the maximum dye penetration.
- Diaproseal > Apexit Plus > MTA Fillapex > Bio-C.
- There was significant difference between the all-sealers group except,
- The difference between MTA Fillapex and BIO-C were non-significant.

## Data Availability

No datasets were generated or analysed during the current study.
